# Polydopamine functionalized dendritic fibrous silica nanoparticles as a generic platform for nucleic acid-based biosensing

**DOI:** 10.1007/s00604-024-06234-2

**Published:** 2024-03-05

**Authors:** Xiaoting Xue, Helena Persson, Lei Ye

**Affiliations:** 1 Division of Pure and Applied Biochemistry, Department of Chemistry, Lund University, 22100 Lund, Sweden; 2 Division of Oncology, Department of Clinical Sciences, Lund University Cancer Center, 22381 Lund, Sweden

**Keywords:** Nanopore, Fluorescence, Biomarker, Quenching

## Abstract

Accurate and rapid detection of nucleic acid sequences is of utmost importance in various fields, including disease monitoring, clinical treatment, gene analysis and drug discovery. In this study, we developed a "turn-on" fluorescence biosensor that enables simple and highly efficient detection of nucleic acid biomarkers. Our approach involves the utilization of 6-carboxyfluorescein modified single-stranded DNA (FAM-ssDNA) as molecular recognition element, along with polydopamine-functionalized dendritic fibrous nanosilica (DFNS). FAM-ssDNA serves as both specific molecular recognition element for the target analyte and reporter capable of transducing a detectable signal through Watson–Crick base pairing. The polydopamine-functionalized DFNS (DFNS@DA) exhibits strong binding to FAM-ssDNA via polyvalent metal mediated coordination leading to effective quenching by fluorescence resonance energy transfer. In the presence of a complementary target sequence, FAM-ssDNA forms hybridized structure and detaches from DFNS@DA, which causes an increased fluorescence emission. The analytical system based on FAM-ssDNA and DFNS@DA demonstrates exceptional sensitivity, selectivity, and rapid response for the detection of nucleic acid sequences, leveraging the high adsorption and quenching properties of DFNS@DA. For the first proof of concept, we demonstrated the successful detection of microRNA (miR-21) in cancer cells using the FAM-ssDNA/DFNS@DA system. Our results highlight the promising capabilities of DFNS@DA and nucleic acid-based biosensors, offering a generic and cost-effective solution for the detection of nucleic acid-related biomarkers.

**Graphical abstract:**

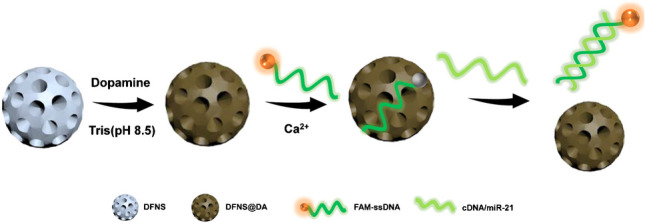

**Supplementary Information:**

The online version contains supplementary material available at 10.1007/s00604-024-06234-2.

## Introduction

Rapid detection and sequencing of nucleic acids have become a fundamental necessity in disease monitoring, clinical treatment, gene analysis and drug discovery [1, 2]. The conventional nucleic acid detection methods including high-throughput sequencing, and reverse transcription polymerase chain reaction have been broadly employed as reliable and gold standard procedures, but these approaches suffer from the restrictions of low sensitivity, complicated operation, time-consuming process, and use of costly equipment, therefore are difficult to implement for routine sample analysis [3, 4]. Developing rapid, cost-effective and scalable nucleic acid detection and quantification methods that are applicable to low income countries and disaster zones are essential [5, 6]. Among the recently developed analytical methods, nanomaterial-based nucleic acid probes represent one of the mainstream directions of technology development [7, 8].

Watson–Crick base pairing between complementary nucleic acid sequences is one of the most important fundamental processes of molecular recognition in vivo, such as during DNA replication and transcription of genetic information [9, 10]. Nucleic acids also play a crucial role in sensing applications by acting as molecular recognition elements to offer high specificity [11, 12], and as reporters to generate a detection signal [13, 14]. The advantages of nucleic acids include their biocompatibility, modular structure, ease of synthesis, and ability to be chemically modified in a sequence-defined manner [15]. Spherical nucleic acids (SNAs) consist of a nanoparticle core that is densely functionalized with nucleic acids [15, 16]. One notable features of SNAs is that the dense and oriented nucleic acids on surface exhibit higher affinity for their complementary sequences in comparison with the free nucleic acids in solution [17].

Dendritic fibrous nanosilica (DFNS), one of the newest members of silica nanomaterials, possesses a unique three-dimensional structure containing centre-radial nanochannels [18, 19]. The high surface area and fast mass transfer properties make DFNS an ideal candidate for preparing SNAs. Surface-functionalization of DFNS is an essential step to prepare DNA-coated SNAs [20, 21]. Dopamine, the protagonist of mussel-inspired bio-adhesive molecule, can oxidize and polymerize spontaneously under alkaline condition to form a thin polydopamine (PDA) coating on various materials. PDA has good biocompatibility [22, 23], and contains abundant functional groups that can facilitate further loading of interesting biomolecules. Moreover, PDA has strong absorption in the whole ultraviolet − visible (UV − vis) light range [24, 25], suggesting its potential use as a generic fluorescence quencher.

**Scheme 1:**
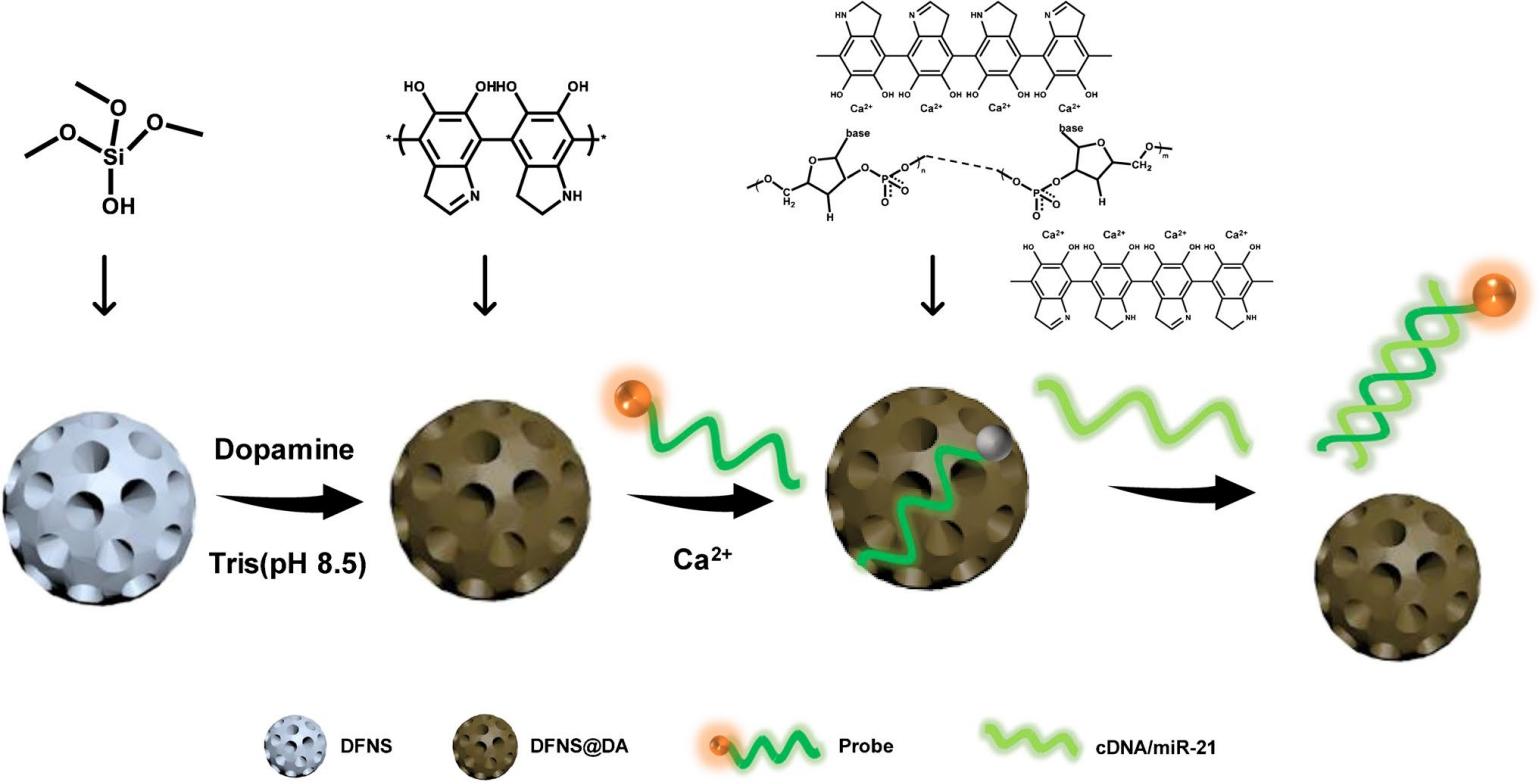
Schematic illustration of the “turn-on” fluorescence sensing platform for nucleic acid detection. The DFNS@DA adsorbs the labelled ssDNA probe and quenches the fluorescence. Hybridization with complementary DNA sequence (cDNA) or microRNA (miR-21) causes the probe to detach from the DFNS@DA to emit fluorescence

In this study, we synthesized DFNS with 4 nm pore size (DFNS-4), DFNS with 20 nm pore size (DFNS-20), and smooth nanosilica (SNS). The nanosilica was functionalized with a thin layer of PDA in order to bind and quench fluorophore-labelled short ssDNA. A 6-carboxyfluorescein-labeled single strand oligodeoxynucleotide (FAM-ssDNA) was designed to recognize the target sequence and act as a fluorescent reporter. The FAM-ssDNA and DFNS@DA are combined to form a fluorogenic complex that can be “turned-on” by exposure to the targeted nucleic acid sequences. As shown in Scheme 1, when the FAM-ssDNA is adsorbed on the DFNS@DA surface, the fluorescence is quenched and the FAM-ssDNA/DFNS@DA system is in the “off” state. In the presence of a complementary target sequence, the FAM-ssDNA hybridizes with the target and detaches from the DFNS@DA, leading to recovery of the fluorescence emission. The target sequence therefore acts as a trigger to “turn-on” the FAM-ssDNA/DFNS@DA biosensor. One unique character of the proposed analytical system is the well-defined pore structure of the DFNS. Because of the small pore size, only the FAM-ssDNA can effectively enter the DFNS@DA to serve as fluorescent sensor. For the first proof of concept, we demonstrate the detection of microRNA (miR-21) in living cells using the proposed analytical system.

## Experimental section

### Materials

Hexadecyl trimethyl ammonium bromide (CTAB), tetraethyl orthosilicate (TEOS), triethanolamine (TEA), sodium salicylate (NaSal), hydrochloric acid (HCl, 37%), ammonia solution (25%), ethyl ether, (3-aminopropyl)-triethoxysilane (APTES), dopamine hydrochloride (DA·HCl), 4-(2-hydroxyethyl)-1-piperazineethanesulfonic acid (HEPES), calcium chloride, magnesium chloride, sodium chloride, human serum albumin (HSA), myoglobin (Mb), ovalbumin (OVA), horse serum, cholera toxin, hydrocortisone and TRI Reagent were purchased from Sigma-Aldrich. Epidermal growth factor (EGF) and insulin were purchased from Thermo Fisher Scientific. Dulbecco’s Modified Eagle’s Medium (DMEM) High Glucose, fetal bovine serum and DMEM: F12 were purchased from HyClone. RNeasy Mini kit was purchased from QIAGEN. RNA 6000 Nano kit was purchased from Agilent Technologies. All buffers and solutions were prepared with purified water from a Milli-Q water purification system (Millipore Corp., Bedford, USA). All HPLC-purified oligonucleotide sequences were synthesized by GenScript. The nucleotide sequences used in this work are given in Table 1.

**Table 1:** Oligonucleotide sequences used in this study

Name	Sequence (5’ to 3’)
Probe	TCAAC ATCAG TCTGA TAAGC TA-FAM
cDNA	TAGCT TATCA GACTG ATGTT GA
miR-21	UAGCU UAUCA GACUG AUGUU GA
R1	CAGAC AAACT CCAAC GA
R2	GATGG GGCAT AATGA GGTGG
R3	AAAAA GATGG GGCAT AATGA
R4	TTAAC CTTTC TCCAT ACGCG GAAGT GAGGT
R5	ACCTC ACTTC CGCGT ATGGA GAAAG GTTAA TAAGA CTTAA CCTTT CTCCA TACGC GGAAG
Let-7a	UGAGG UAGUA GGUUG UAUAG UU
Let-7e	UGAGG UAGGA GGUUG UAUAG UU
miR-122	UGGAG UGUGA CAAUG GUGUU UG
miR-143	UGAGA UGAAG CACUG UAGCU C
miR-144	UACAG UAUAG AUGAU GUACU

### Instruments and characterization

Dynamic light scattering (DLS) (Zetasizer Nano ZS, Malvern Instruments, UK), a FT-IR spectrometer (Nicolet iS5, Thermo Fisher Scientific Inc., Waltham, USA), Cary Eclipse fluorescence spectrophotometer (Agilent Technologies, USA), scanning electron microscope (JSM-6700F, JEOL, Japan), transmission electron microscope (JEM-1400Plus, JEOL, Japan). Nitrogen adsorption − desorption measurements were carried out on an automated surface area analyzer (Model ASAP 2400 of Micrometrics Co., Inc., USA). Thermogravimetric analysis was carried out using a TGA Q500 Thermogravimetric Analyzer (TA Instruments). Time-resolved fluorescence experiments were conducted using a lifetime fluorescence spectrometer EasyLife X (Horiba, USA). Automated electrophoresis was performed using 2100 Bioanalyzer (Agilent Technologies, USA).

### Synthesis of polydopamine functionalized dendritic fibrous silica (DFNS@DA)

Preparation of nanosilica (NS, including SNS, DFNS-4 and DFNS-20) are described in the supporting information.

Dry NS were modified with PDA by oxidative self-polymerization of DA. Briefly, 20 mg of NS were added to 10 mL of Tris buffer (10 mM, pH 8.5) and sonicated until the NS dispersed evenly in the buffer. DA·HCl (40 mg) was dissolved in 10 mL of Tris buffer (10 mM, pH 8.5) and then added to the NS suspension. The reaction mixture was stirred for 16 h at room temperature. The products were collected by centrifugation, washed with water several times, and finally stored in water (20 mL) for further experiments.

### Detection of cDNA and miR-21

The FAM-ssDNA probe (10 μL of 20 μM solution) was mixed with 50 μL of NS@DA in HEPES buffer (10 mM, pH 7.4) and 2 mM CaCl_2_ (total volume 1 mL) for 30 min at room temperature. The obtained probe − NS@DA complex solution (180 μL) was mixed with 20 μL of cDNA or miR-21 at different concentrations and shaken for 30 min at room temperature. The fluorescence intensity of the samples was measured directly with the fluorometer. Other fluorescence assays are described in the supporting information.

### Cell culture and RNA extraction

The MCF7 and MCF 10A cell lines were obtained from American Type Culture Collection (ATCC) and cultured at 37 °C, 5% CO_2_ in a humidified atmosphere. MCF7 were cultured in Dulbecco’s Modified Eagle’s Medium (DMEM) High Glucose with 10% fetal bovine serum and 10 µg/mL insulin. MCF 10A were cultured in DMEM: F12 with 5% horse serum 10 µg/mL insulin, 20 ng/mL EGF, 100 ng/mL cholera toxin and 500 ng/mL hydrocortisone. Cells were harvested in TRI Reagent for extraction of total RNA according to the manufacturer’s instructions. Depletion of small RNAs was performed with the RNeasy Mini kit using the RNA clean-up protocol and removal of the small RNA fraction was confirmed using a 2100 Bioanalyzer with the RNA 6000 Nano kit.

## Results and discussion

### Characterization of DFNS@DA

**Fig. 1:**
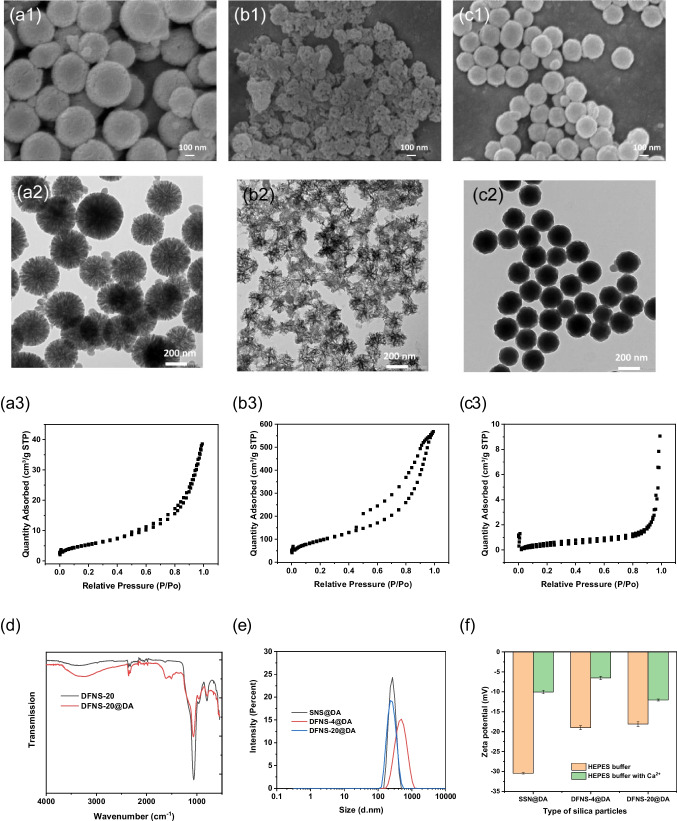
SEM and TEM images, N_2_ sorption isotherms of (**a**) DFNS-4@DA, (**b**) DFNS-20@DA, (**c**) SNS@DA, (**d**) FT-IR spectra of DFNS-20 and DFNS-20@DA, (**e**) the hydrodynamic diameter of SNS@DA, DFNS-4@DA and DFNS-20@DA, (**f**) Zeta potential of SNS@DA, DFNS-4@DA and DFNS-20@DA in buffer with or without Ca^2+^

The SEM and TEM images in Fig. S1 show the monodispersed DFNS-4 and DFNS-20 with a diameter of ∼400 nm and ∼200 nm, respectively. Both DFNS-4 and DFNS-20 have a central-radial dendritic porous structure, and DFNS-20 has larger pore size than DFNS-4 (Fig. S1a1-a2, b1-b2). The non-porous SNS has a smooth surface and is spherical with a diameter of ∼200 nm (Fig. S1c1-c2). Dynamic light scattering (DLS) analysis shows that the hydrodynamic diameters of SNS, DFNS-4 and DFNS-20 were 249.4, 468.6 and 242.4 nm, respectively (Table 2). The BET surface areas of DFNS-4, DFNS-20 and SNS were 460.6, 783.5 and 16.7 m^2^/g, respectively (Fig. S1a3, b3, c3 and Table 2). The pore size of DFNS-4, calculated from the desorption isotherm using the BJH method, was 4 nm. The size of the predominant pores in DFNS-20 was found to be 20 nm (Fig. S1a4, b4 and Table 1). After coating PDA on the surface of the nanoslica for 16 h, the BET surface areas of DFNS-4@DA, DFNS-20@DA and SNS@DA changed to 20.5, 359 and 2.41 m^2^/g, respectively (Fig. 1a3, b3, c3 and Table 2). The mesopores in DFNS-4@DA was therefore filled up with the PDA polymer, and the predominant mesopores in DFNS-20@DA became smaller (15 nm) after coating with PDA (Fig. S2a1, b1 and Table 2). As revealed by the SEM and TEM images (Fig. 1a1-a2 and b1-b2), DFNS-4@DA and DFNS-20@DA have no obvious change in particle sizes after coating with PDA, suggesting that the PDA was coated predominantly on the inner surface of the mesopores. For SNS@DA, the particle surface became rougher due to the attachment of the PDA layer on the core particles (Fig. 1c1&2).

**Table 2:** Physical properties of NS and NS@DA

NS	D_DLS_^a)^		PDI^b)^		S_BET_^c)^		PD_BET_^d)^		W_TGA_^e)^	
	NS	NS@DA	NS	NS@DA	NS	NS@DA	NS	NS@DA	NS	NS@DA
SNS	249.4	262.2	0.034	0.039	16.73	2.41	/	/	6.0	32.7
DFNS-4	468.6	473.5	0.046	0.16	460.57	20.47	4	4	11.6	37.9
DFNS-20	242.4	252.4	0.26	0.087	783.48	35.9	6/20	4/15	5.6	29.0

^a)^ Hydrodynamic diameter (nm); ^b)^ Polydispersity index; ^c)^ Specific BET surface area (m^2^/g); ^d)^ Pore diameter (nm); ^e)^ Weight loss in TGA analysis (%)

The TGA results reveal that the mass ratio of PDA in SNS@DA, DFNS-4@DA and DFNS-20@DA were 26.7%, 25.4% and 23.4%, respectively (Fig. S2a2, b2, c2 and Table 2), indicating the successful synthesis of PDA layer on the nanosilica surfaces. The amount of PDA formed on the non-porous silica nanoparticles was slightly higher than on the mesoporous particles. DLS analysis reveals that the hydrodynamic diameters of SNS@DA, DFNS-4@DA and DFNS-20@DA were 262.2, 473.5 and 252.4 nm, respectively (Fig. 1e and Table 2). Compared with the core particles before coating, the thickness of the PDA layer was estimated to be around 10 nm. FTIR spectra of DFNS-20 and DFNS-20@DA were recorded to provide qualitative information of the PDA coating (Fig. 1d). Compared to DFNS-20, DFNS-20@DA displayed new characteristic adsorption bands. The band at 1615 cm^−1^ is attributed to the N − H stretching, and the adsorption bands at 1506 and 1357 cm^−1^ are assigned to benzene ring C − C vibration [26]. A visible change in colour from white to brown was also observed from the particles after the PDA coating (Fig. S3). These results confirmed the presence of the PDA coating on the silica nanoparticles.

Good dispersibility of particles in water is crucial for successful detection of analytical target since the fluorescent quenching and recovery are to be measured in solution. The rate of particle sedimentation was determined by monitoring the change of absorbance (Abs) at 600 nm for PDA nanoparticles and DFNS-20@DA suspension. As shown in Fig. S4, the PDA nanoparticles exhibited significantly faster sedimentation than DFNS-20@DA, indicating that the silica skeleton in DFNS-20@DA contributed to the much-improved dispersibility. The low polydispersity indices (PDIs) of 0.040, 0.16 and 0.087 indicate that the PDA-coated SNS@DA, DFNS-4@DA and DFNS-20@DA can form stable and non-aggregating suspensions in water (Table 1). The Zeta potential of these PDA-coated nanoparticles in buffer were measured and found to be -30.47, -19.00 and -18.08 mV, respectively (Fig. 1f). Since a ζ potential of <  − 25 mV is normally considered appropriate to achieve a high colloidal stability [27], the PDA-coated nanosilica used in this work have satisfactory water dispersibility.

### Adsorption of ssDNA by DFNS@DA and mechanism of fluorescence quenching

The purpose of functionalizing DFNS with PDA is to enable adsorption of FAM-ssDNA to achieve effective quenching. The thickness of the PDA coating can be controlled by adjusting the polymerization time and reaction conditions [28, 29]. In this study, the PDA functionalization on the surface of DFNS was achieved through self-polymerization of dopamine at alkaline pH (∼8.5). We measured the quenching efficiency of DFNS-4@DA obtained by using different polymerization times. Fig. S5 shows that after 2 h of polymerization, the obtained DFNS-4@DA achieved a quenching efficiency (QE) of more than 95%. Increasing the polymerization time after 2 h did not significantly affect the QE. Therefore, a polymerization time of 2 h is sufficient to prepare PDA-coated DFNS-4 that is suitable to bind ssDNA for effective quenching.

**Fig. 2:**
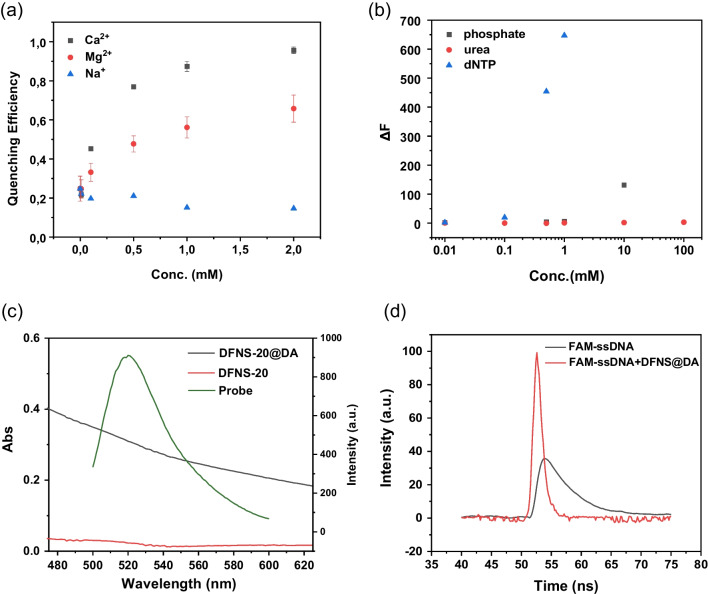
**a** Quenching efficiency of DFNS-20@DA toward FAM-ssDNA in the presence of Ca^2+^, Mg^2+^ and Na^+^ in 10 mM HEPES buffer. **b** FAM-ssDNA desorption from DFNS-20@DA in the presence of different concentrations of inorganic phosphate and dNTPs. **c** Fluorescence emission of FAM-ssDNA, UV–vis spectra of DFNS-20@DA and DFNS-20. **d** Time-resolved fluorescence emission of FAM-ssDNA and FAM-ssDNA mixed with DFNS@DA

DNA is a negatively charged polyanion due to its phosphate backbone. The negative surface charge of PDA is expected to influence the interaction between DNA and PDA. The presence of Na^+^ is known to reduce the charge repulsion and promote DNA adsorption on negatively charged surfaces, e.g. on negatively charged gold nanoparticles [30, 31] and graphene oxide [32]. However, as shown in Fig. 2a, increasing the concentration of Na^+^ from 0 to 2 mM did not result in fluorescence quenching. Therefore, the attractive force between FAM-ssDNA and the PDA-coated DFNS-20@DA needs to be increased. Considering that the catechol groups of dopamine can interact strongly with divalent metal ions, we tested the effect of different concentrations of Ca^2+^ and Mg^2+^ on the efficiency of quenching. The quenching efficiency increased as the concentration of Ca^2+^ and Mg^2+^ increased. At the same concentration, Ca^2+^ was found to be even more effective than Mg^2+^ for ssDNA adsorption and quenching. With 2 mM Ca^2+^, the QE reached 98%. Therefore, the buffer containing 2 mM Ca^2+^ was selected as the solvent for all subsequent quenching experiments. Figure 1f shows the Zeta potential of the PDA-coated nanosilica (NS@DA) measured in buffer with and without Ca^2+^. Although the surface charge of NS@DA became less negative due to the presence of 2 mM Ca^2+^, it still remained negative. Thus, the improved adsorption of ssDNA by Ca^2+^ is not completely caused by neutralization of charge repulsion.

Several other types of interactions may force the ssDNA probe to adsorb onto DFNS@DA, including hydrogen bonding, π − π stacking, and Lewis’s acid − base interactions. To determine whether hydrogen bonding had any impact on ssDNA binding, urea was selected as a potent hydrogen-bonding disruptor to test its effect on ssDNA binding [33]. Since the nucleobases and phosphate backbone are the two main components in FAM-ssDNA, we also tested ssDNA binding in the presence of inorganic phosphate and deoxynucleotide triphosphates (dNTPs). As shown in Fig. 2b, addition of 100 mM urea caused almost no DNA to detach from DFNS-20@DA, indicating that hydrogen bonding is not the main driving force for ssDNA adsorption. The fluorescence signal of the FAM-ssDNA/DFNS-20@DA mixture remained at the background level after addition of inorganic phosphate up to 1 mM, then began to increase when the concentration of inorganic phosphate reached 10 mM. The addition of dNTPs appeared to have a great impact on ssDNA binding. When the concentration of dNTPs changed from 0.1 mM to 0.5 mM, the fluorescence intensity increased by more than 20 folds. Therefore, the nucleobases in the ssDNA play a more critical role than the phosphate backbone for ssDNA adsorption on the PDA coating.

The UV − vis spectrum of DFNS@DA showed strong absorption from 500 to 600 nm that overlapped with the fluorescence emission band of FAM-ssDNA. In contrast, the uncoated DFNS had almost no UV–vis adsorption in the same range (Fig. 2c). To determine the fluorescence lifetime of FAM-ssDNA with and without DFNS@DA, we measured time-resolved fluorescence spectra of FAM-ssDNA, with and without DFNS@DA (Fig. 2(d)). The lifetime of FAM-ssDNA was determined to be 4.5 ns, which changed to 0.15 ns after DFNS@DA was introduced. Thus, the fluorescence quenching is most likely caused by the Förster resonance energy transfer (FRET) between the “donor” (FAM) and the “acceptor” (PDA) [34].

Based on these experimental results, it is plausible that the interaction between FAM-ssDNA and DFNS@DA is primarily driven by the π − π stacking interaction between the exposed nucleobases in the ssDNA and the quinone-related structures in the PDA coating of DFNS@DA. The presence of Ca^2+^ is likely to facilitate ssDNA adsorption through interactions with the DNA bases, which further enhances the π − π stacking interaction [35]. The phosphate (a hard Lewis base) in the backbone of the ssDNA may also contribute to the binding to DFNS@DA by interacting with Ca^2+^ (a hard Lewis acid) [36]. As a result of the adsorption, the distance between the bound FAM-ssDNA and the PDA coating is shortened, thereby enabling an effective fluorescence quenching.

### Hybridization-triggered fluorescence emission

**Fig. 3:**
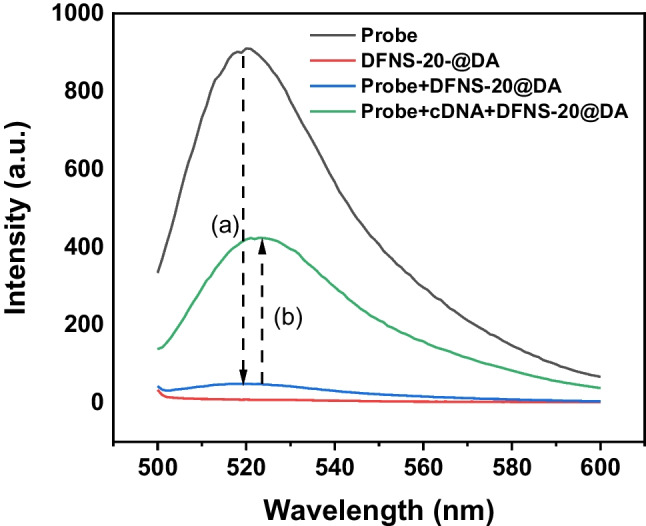
Fluorescence spectra of FAM-ssDNA, DFNS-20@DA, FAM-ssDNA + DFNS-20@DA and FAM-ssDNA + cDNA + DFNS-20@DA. FAM-ssDNA: 200 nM; cDNA: 200 nM; DFNS-20@DA: 10 µL (0.01 mg/mL). Total volume of measurement is 200 µL and in buffer (10 mM HEPES, pH 7.4, 2 mM CaCl_2_)

To investigate the possibility of recovering fluorescence by complementary base pairing, we measured the fluorescence emission of a mixture of DFNS-20@DA and FAM-ssDNA, before and after addition of a complementary ssDNA (cDNA). As shown in Fig. 3, the fluorescence of FAM-ssDNA (200 nM) was quenched by DFNS-20@DA (arrow a in Fig. 3). After addition of the cDNA (200 nM), nearly 50% of the fluorescence was recovered (arrow b in Fig. 3), indicating that hybridized dsDNA formed and detached from DFNS-20@DA. When the uncoated DFNS-20 was tested under the same condition, the nanosilica was not able to quench the fluorescence from FAM-ssDNA (Fig. S6). These results demonstrate the high sensitivity and specificity of the DFNS@DA-based “turn-on” fluorescence sensor.

### Analytical performance of DFNS@DA-based fluorescence sensor

**Fig. 4:**
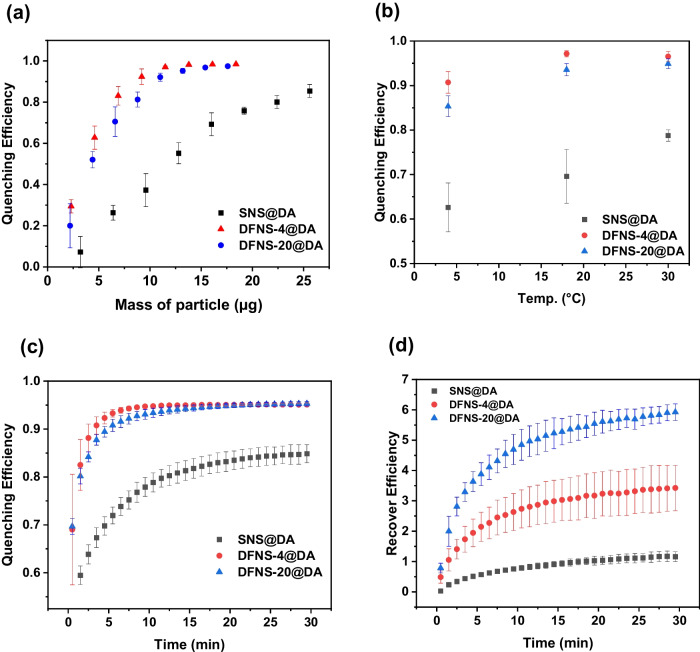
**a** Quenching efficiency of NS@DA at different NS@DA content (FAM-ssDNA: 200 nM; NS@DA: 2–18 µL; quenching time: 30 min; temperature: 18 °C), **b** quenching efficiency of NS@DA at 4 °C, 18 °C and 30 °C (FAM-ssDNA: 200 nM; NS@DA: 10 µL; quenching time: 30 min.), **c** kinetic of Quenching efficiency of NS@DA (FAM-ssDNA: 200 nM; NS@DA: 10 µL; quenching time: 30 min, temperature: 18 °C), **d** Kinetic of recovery efficiency of NS@DA (FAM-ssDNA: 200 nM; NS@DA: 10 µL; quenching time: 30 min, temperature: 18 °C.). Total volume of measurement is 200 µL and in buffer (10 mM HEPES, pH 7.4, 2 mM CaCl_2_)

The efficiency of fluorescence quenching is influenced by the concentration of the NS@DA and the incubation temperature because the concentration and temperature affect the adsorption of the FAM-ssDNA probe on the NS@DA. Figure 4a shows that the QE increased with increasing nanoparticle content. When 10 µL of DFNS@DA was used, the QE reached to more than 90%. For the PDA-coated nonporous nanosilica, the QE was only 85% with 16 µL of SNS@DA. This result confirms that the PDA-coated DFNS is a more effective quencher than the coated nonporous silica. In Fig. 4b, the QE of DFNS@DA was measured at three different temperatures. The QE at 18 °C and 30 °C were almost the same and were slightly higher than at 4 °C. For SNS@DA, the QE increased with the incubation temperature, suggesting that on the nonporous particles the ssDNA adsorption is more entropy driven. Figure 4c shows the kinetics of fluorescence quenching of the different PDA-coated nanosilica. FAM-ssDNA was quenched much more rapidly after being exposed to DFNS@DA than SNS@DA, and the quenching by the DFNS@DA reached equilibrium within 10 min. In Fig. 4d, the cDNA-triggered fluorescence recovery (recovery efficiency, RE) was measured at different times. Between the two types of DFNS@DA, DFNS-20@DA exhibited higher recovery efficiency (RE) than DFNS-4@DA, although both had similar quenching kinetics. In contrast to the PDA-coated mesoporous nanoparticles, the PDA-coated nonporous particle SNS@DA exhibited poorer fluorescence recovery.

**Fig. 5:**
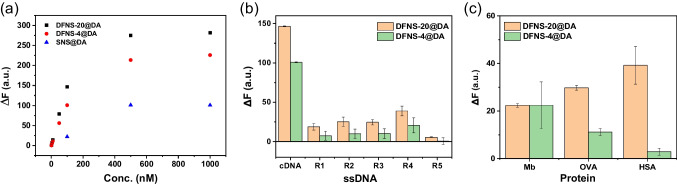
**a** Fluorescence change of DFNS@DA/FAM-ssDNA complex for the detection of cDNA. **b** Fluorescence signal observed from DFNS@DA/FAM-ssDNA complex triggered by 100 nM of different ssDNA sequences. **c** Fluorescence signal observed from DFNS@DA/FAM-ssDNA complex caused by 100 µg/mL of different proteins

Based on the effect of temperature and the kinetics of fluorescence quenching and recovery, we selected room temperature (18 °C), 20 min incubation for fluorescence quenching and 30 min for fluorescence recovery in subsequent experiments to detect cDNA. The DFNS@DA-based “turn-on” fluorescence sensors show increasing fluorescence signal when cDNA concentration changed from 1 to 1000 nM. For SNS@DA-based sensor, the fluorescence signal started to increase at cDNA concentration of 100 nM (Fig. 5a). Therefore, the DFNS@DA-based sensors have a wider detection range.

Biological samples contain various substances, such as proteins and random non-target ssDNA, which could potentially interfere with the adsorption and hybridization-triggered release of FAM-ssDNA, resulting in false signals. ssDNA of different lengths and mismatch sequences (R1-R5) were first selected to verify the selectivity of the FAM-ssDNA/DFNS@DA system. As shown in Fig. 5b, all the non-target ssDNA produced very weak signal (Fig. 5b). Furthermore, we evaluated the interference of some representative proteins including Mb (16 kDa, 2.1 × 3.5 × 4.4 nm), OVA (42.7 kDa, 7 × 4.5 × 5 nm), and HSA (66 kDa, 12 × 4 × 4 nm). Figure 5c shows that the proteins produced some weak fluorescence signal, with Mb giving the same signal between DFNS-4@DA and DFNS-20@DA particles. Both OVA and HSA produced weaker signal on DFNS-4@DA than DFNS-20@DA, most probably due to the smaller pores in DFNS-4@DA, which prevented the large proteins from entering the nanochannels of the DFNS. Therefore, DFNS-4@DA compared more favourably than DFNS-20@DA for resisting protein-induced dissociation of the ssDNA probe.

### Detection of miRNA-21

**Fig. 6:**
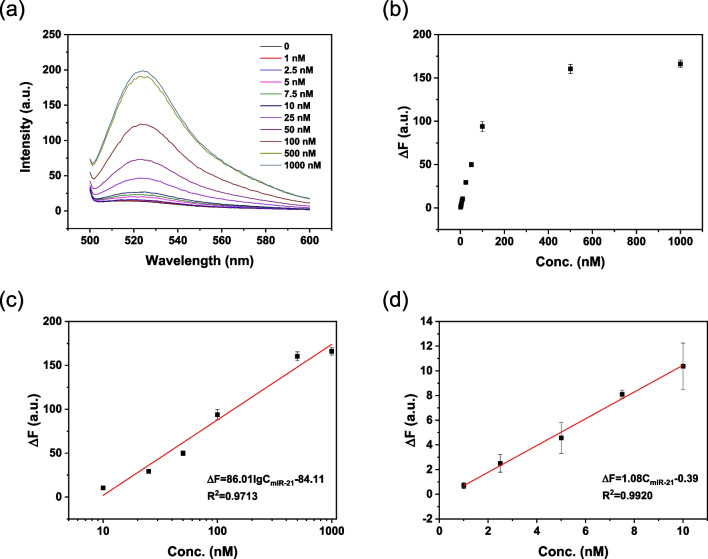
Analytical performance of DFNS-4@DA for detection of miR-21. **a** Fluorescence emission of FAM-ssDNA/DFNS-20@DA complex triggered by miR-21 at different concentrations. **b** ΔF versus the concentration of miR-21. **c** Linear correlation between ΔF and lgC_miR-21_. **d** Linear correlation between ΔF and C_miR21_

We then explored the FAM-ssDNA/DFNS@DA complex for detection of microRNA. microRNAs are small noncoding RNAs and play a significant role in regulating gene expression, making them valuable biomarkers for diseases such as cancer. miR-21 has been identified as an important marker for oncogene that is overexpressed in various cancers, and detection of miR-21 is essential for tumor diagnostics [37, 38]. The fluorescence emission of FAM-ssDNA/DFNS-4@DA triggered by miR-21 is shown in Fig. 6a. The fluorescence intensity increased with increasing concentration of miR-21 in the range of 1–1000 nM (Fig. 6b). In the semi-logarithmic graph (Fig. 6c), the ΔF was linearly related to the logarithm of miR-21 concentration in the range of 10 nM to 1000 nM, with a linear equation of ΔF = 125.02 logC_miR-21_—132.8 (R^2^ = 0.9660) and a LOD of 0.53 nM. The fluorescence intensity change (ΔF) increased linearly with miR-21 in the range from 1 to 10 nM. The linear equation of response is ΔF = 1.38C_miR-21_—1.20 (R^2^ = 0.9633) (Fig. 6d). Detection of miR-21 using DFNS-20@DA was also investigated and the result is shown in Fig. S7. The performance of miR-21 detection using DFNS-20@DA is similar to DFNS-4@DA. Table S1 shows that the analytical performance of DFNS@DA is comparable to other solid phase quenchers when used in “turn-on” fluorescent biosensors for DNA and miRNA. Notably, the method to prepare the DFNS@DA quencher is the most straightforward.

**Fig. 7:**
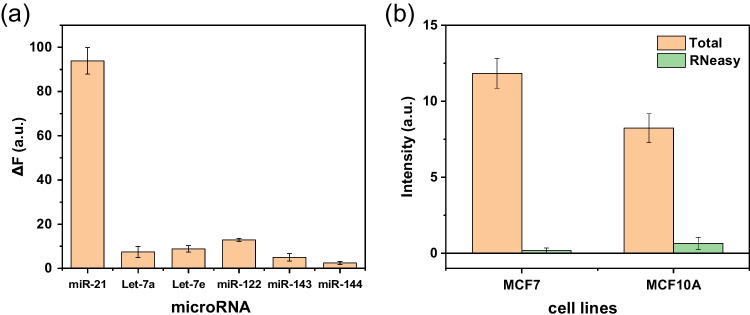
**a** Selectivity analysis for the detection of miR-21. **b** Fluorescence change of FAM-ssDNA/DFNS-4@DA complex for the detection of miR-21 in total and size-selected (RNeasy) RNA from MCF7 and MCF 10A cells

The selectivity of FAM-ssDNA/DFNS@DA complex for miR-21 detection was further confirmed using five common miRNAs as references: miR-122, miR-143, miR-let-7a, miR-let-7e and miR-144 (Fig. 7). None of these miRNAs was able to recover the fluorescence of FAM-ssDNA. Effective fluorescence recovery occurred only when FAM-ssDNA/DFNS-4@DA was exposed to the target miR-21 (Fig. 7a). To evaluate the practicality of the proposed method for detection of miR-21, RNA from the two human cell lines MCF7 (breast cancer) and MCF 10A (immortalized normal breast cells) was used in the subsequent in vitro experiments. As a negative control, we used RNA from both cell lines that had been extracted with the RNeasy kit which removed virtually all small RNAs. The RNeasy-treated samples generated a very weak fluorescence signal (Fig. 7b). Therefore, the remaining larger RNA molecules were unable to recover the fluorescence of FAM-ssDNA. In contrast, total RNA from MCF7 and MCF 10A generate obvious fluorescence signal. The fluorescence signal for the MCF7 cancer cell line was higher than for the normal MCF 10A cells, consistent with the higher expression of miR-21 in MCF7 vs MCF 10A. The result indicated that the FAM-ssDNA/DFNS-4@DA complex can differentiate between cancer and normal cells, which was similar to the result of the literatures [35, 38].

## Conclusions

In this work, a “turn-on” fluorescence biosensor based on FAM-ssDNA/DFNS@DA complex was developed for rapid and straightforward detection of nucleic acid sequences. The combination of the unique structure of DFNS and the fluorescence quenching by PDA coating allowed us to construct the fluorescence sensor that can tolerate various interfering molecules in biological samples. The release of the fluorophore labeled ssDNA upon target recognition leads to effective fluorescence recovery, enabling rapid and specific detection of the nucleic acid targets. The proposed method for DNA/RNA analysis can be implemented using the generic platform of PDA-coated DFNS without complicated material synthesis. Given that short ssDNA sequences are widely used as labels and probes in biological assays, the analytical platform demonstrated in this work can be combined with antibodies and aptamers to detect a wide variety of biological targets including cellular metabolites, proteins and whole cells.

### Supplementary Information

Below is the link to the electronic supplementary material.

Supplementary file1 (PDF 859 KB)

## Data Availability

All data generated or analysed during this study are included in this published article and its supplementary information files.
