# Supplementary material for: Polydopamine functionalized dendritic fibrous silica nanoparticles as a generic platform for nucleic acid-based biosensing
Source: Mikrochim Acta. 2024 Mar 5;191(4):180. doi: 10.1007/s00604-024-06234-2 (PMC10914921; doi:10.1007/s00604-024-06234-2)
Supplement: Supplementary file 1 — Supplementary file1 (PDF 859 KB) [file 604_2024_6234_MOESM1_ESM.pdf]

Electronic Supplementary Information (ESI)

Polydopamine functionalized dendritic fibrous silica nanoparticles as a generic platform for nucleic acid-based biosensing

*Xiaoting Xue [a], Helena Persson [b], Lei Ye [a]**

[a] Division of Pure and Applied Biochemistry, Department of Chemistry, Lund University, Box 124, 22100 Lund, Sweden

[b] Division of Oncology, Department of Clinical Sciences, Lund University Cancer Centre, 22381 Lund, Sweden

Corresponding author: Lei Ye, Email: lei.ye@tbiokem.lth.se Tel.: +46 46 2229560

Experimental section

Preparation of SNS, DFNS-4 and DFNS-20

SNS were synthesized using a one-step Stöber procedure.[1] A solution containing 100 mL of methanol, 33 mL of water, and 22.4 mL of ammonia (25%) was stirred using a magnetic stirrer in a 1000 mL glass beaker. A mixture of 130 mL of methanol containing 13.8 mL of TEOS was then rapidly added to the solution. The reaction mixture was stirred at room temperature for 8 h. The SNS were isolated by centrifugation, washed with water and methanol several times, and dried in a vacuum desiccator.

DFNS-4 was synthesized by following a sol-gel reaction[2], with slight modification. In a 100 mL round-bottom flask, 70 mL of water, 1 mL of aqueous ammonia (25%), 20 mL of ethyl ether, and 10 mL of ethanol were added and vigorously stirred with a magnetic stirrer at room temperature for 30 min, 500 mg of CTAB was then added to the mixture. After 30 min, a mixture of 2.5 mL of TEOS and 0.1 mL of APTES was added quickly into the above-mentioned mixture. The reaction mixture was stirred vigorously at room temperature for 4 h. Next, 1 mL of HCl (37%) was added to quench the reaction. The nanoparticles were isolated by centrifugation, washed with water and ethanol three times, and resuspended in 120 mL of ethanol by sonication, followed by addition of 15 mL of HCl (37%). The mixture was stirred at 70 °C for 24 h, and then the nanoparticles were isolated by centrifugation and washed with ethanol three times to remove the surfactant CTAB from DFNS. The DFNS-4 was collected by centrifugation and dried in a vacuum desiccator.

DFNS-20 were synthesized following the procedure reported by Yu et al.[3] In a 250 mL round-bottomed flask, 100 mL of water and 0.272 g of TEA were added and stirred with a magnetic stirrer for 30 min at 80°C. Then, 1.52 g of CTAB and 0.672 g of NaSal were added to the mixture and stirred vigorously for 1 hour. After adding 16 mL of TEOS and 1 mL of ethanol, the reaction mixture was stirred vigorously for 2 h. The obtained nanoparticles were separated by centrifugation, washed with ethanol (three times), and then re-suspended in a mixture of HCl (37%) and methanol (1: 6 volume ratio) to remove CTAB. The particle suspension was stirred at 65 °C before the particles are separated and transferred into a new solution every 12 h. This process was repeated three times. Finally, the DFNS-20 were washed thoroughly with methanol and dried in a vacuum desiccator at room temperature overnight.

Fluorescence assays

The fluorescence spectra for probe were obtained at excitation and emission wavelengths of 488 and 518 nm, respectively. The following parameters were set for fluorescence measurement: a photomultiplier tube voltage of medium, a scan speed of medium, and excitation and emission slits of 10 nm. Fluorescence quenching efficiency (QE) was calculated using the formula QE = $1 - F_M/F_0$, where F_M and F_0 represent the fluorescence intensities of probe in the presence and absence of NS@DA, respectively. Fluorescence recovery efficiency (RE) was calculated using the formula RE = $F_T/F_M - 1$, where F_T and F_M represent the fluorescence intensities of probe in the presence and absence of cDNA after NS@DA addition, respectively.

For the effect of the amounts of NS@DA, 2-16 μL of NS@DA solution was added to HEPES buffer (10 mM, pH 7.4) containing 2 mM $CaCl_2$ (total volume 198 μL), then 2 μL of probe (20 μM) was added. After a 30 min incubation time at room temperature, the mixture was subjected to fluorescence measurements. For the effect of incubation temperature, 10 μL of SNS@DA solution was added to HEPES buffer (10 mM, pH 7.4) containing 2 mM $CaCl_2$ (total volume 198 μL), then 2 μL of probe (20 μM) was added. After a 30 min incubation time at different temperatures, the mixture was subjected to fluorescence measurements.

For the kinetic studies of the fluorescence intensities of quenching and recovery, 10 μL of NS@DA solution was added to 188 μL HEPES buffer (10 mM, pH 7.4) containing 2 mM $CaCl_2$, the mixture was transferred to a cuvette. After adding 2 μL of probe (20 μM), the fluorescence spectrum of the particle suspension was recorded every 0.5 min for 30 min. After this step, 2 μL of cDNA (20 μM) was added to the cuvette, and the fluorescence spectrum was recorded every 0.5 min for 30 min.

For effect of metal ions for quenching, 10 μL of DFNS-20@DA solution was added to HEPES buffer (10 mM, pH 7.4) containing different concentration of $CaCl_2$, $MgCl_2$ and NaCl (total volume 198 μL), then 2 μL of probe (20 μM) was added. After a 30 min incubation time at room temperature, the mixture was subjected to fluorescence measurements.

For displacement studies, the 10 μL of 20 μM probe was blended with 50 μL of DFNS-20@DA in HEPES buffer (10 mM, pH 7.4) containing 2 mM $CaCl_2$ (total volume 1 mL) for 30 min at room temperature. Then, the probe – DFNS-20@DA complex solution (180 μL) was mixed with phosphate, urea and dNTP (20 μL), and shaken for 30 min at room temperature. For selectivity studies, the probe − DFNS@DA complex solution (180 μL) was

mixed with Mb, OVA and HSA (1 mg/mL, 20 μL) or R1-5 (20 mL, 1000 nM), and shaken for 30 min at room temperature.

Supplementary Figures

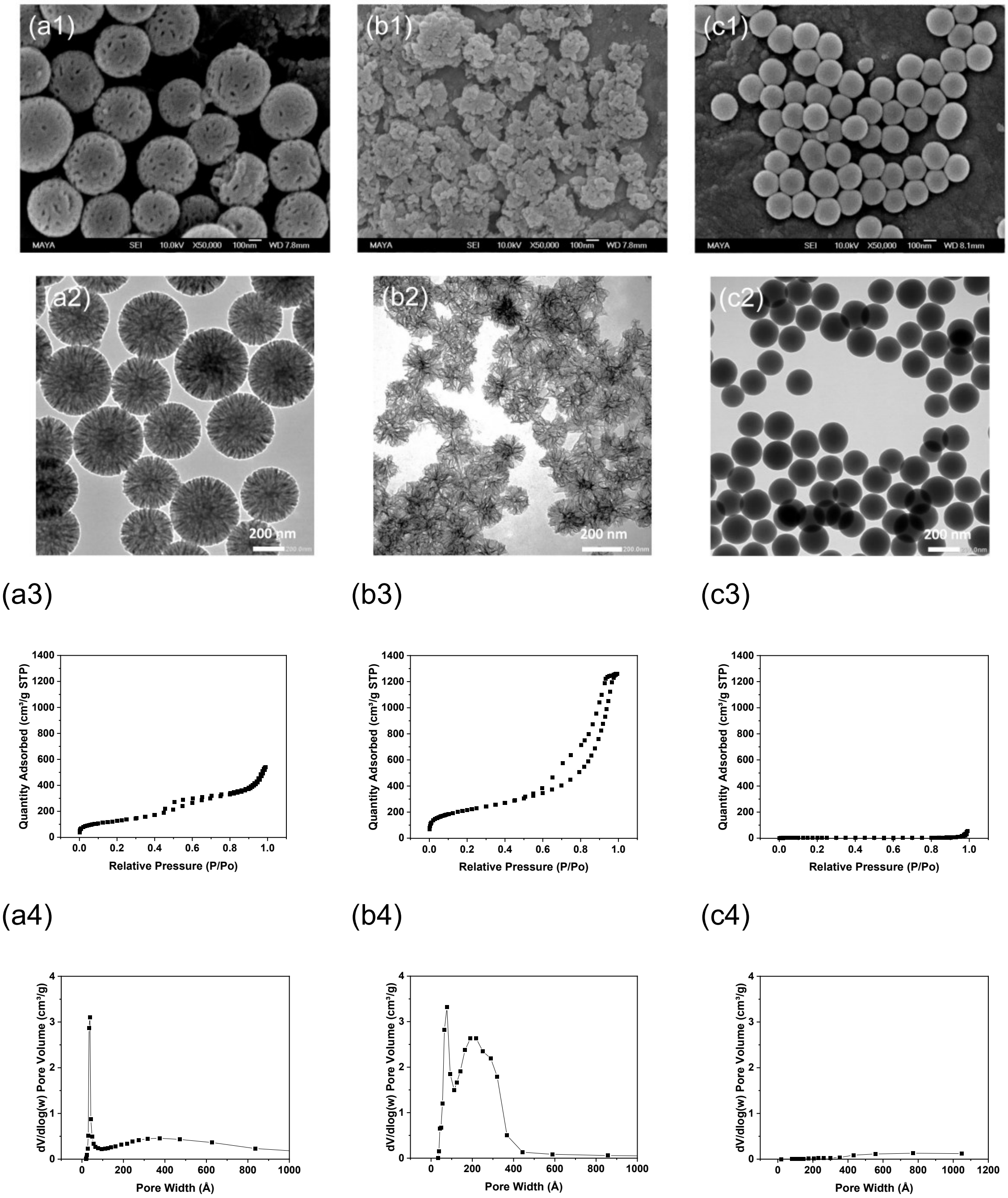

Fig. S1. SEM and TEM images, N_2 sorption isotherms and size distribution of (a) DFNS-20, (b) DFNS-4, and (c) SNS.

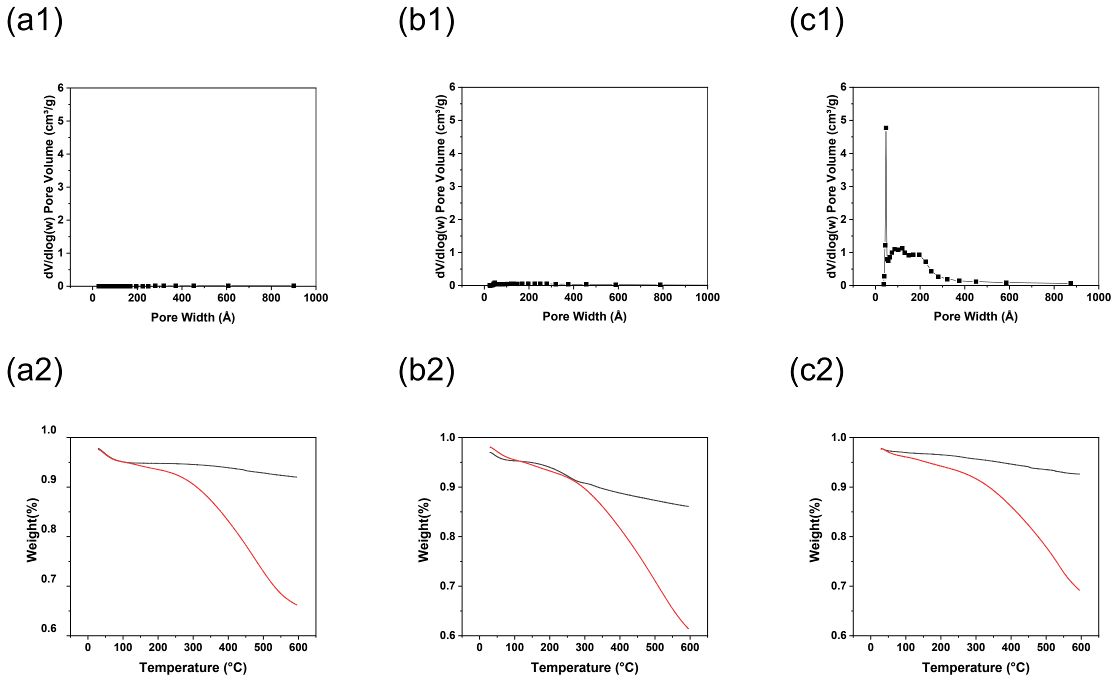

Fig. S2. (a1-c1) Pore size distribution of SNS@DA (a1), DFNS-4@DA (b1), and DFNS-20@DA (c1). (a2-c2) TGA curves of SNS and SNS@DA (a2), DFNS-4 and DFNS-4@DA (b2), DFNS-20 and DFNS-20@DA (c2).

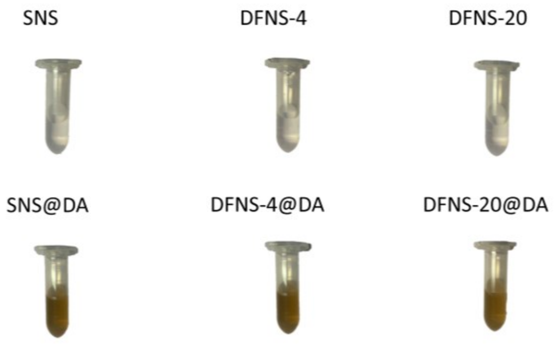

Fig. S3. Photographs of NS and NS@DA (0.1 mg/mL) in water

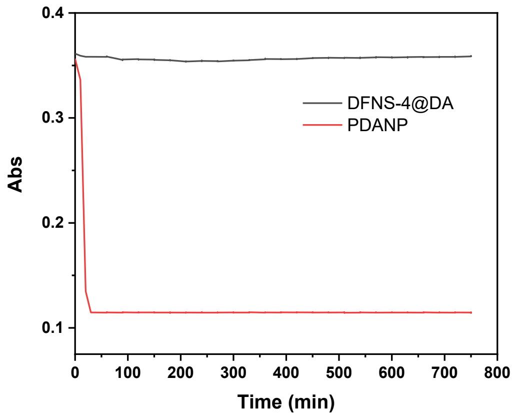

Fig. S4. Absorbance (Abs) of PDA nanoparticles and DFNS-20@DA suspension monitored at 600 nm.

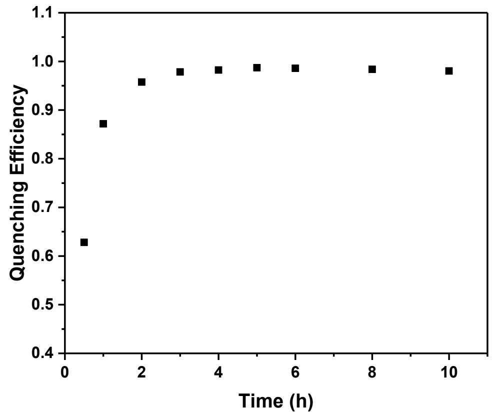

Fig. S5. Quenching efficiency of DFNS-4@DA synthesized using different polymerization times toward FAM-ssDNA.

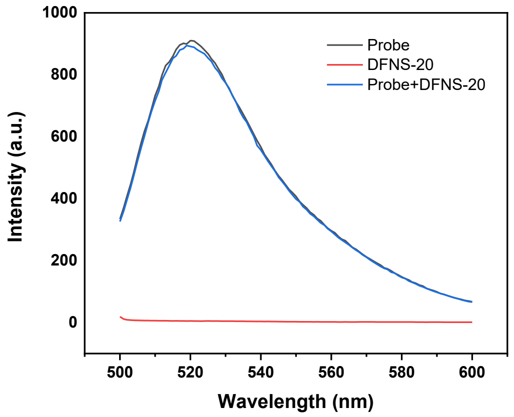

Fig. S6. Fluorescence spectra of probe, DFNS-20 and probe+DFNS-20. Probe: 200 nM; cDNA: 200 nM; DFNS-20@DA: 0.01 mg/mL. Total volume of measurement is 200 μL and in buffer (10 mM HEPES, pH 7.4, 2 mM $CaCl_2$).

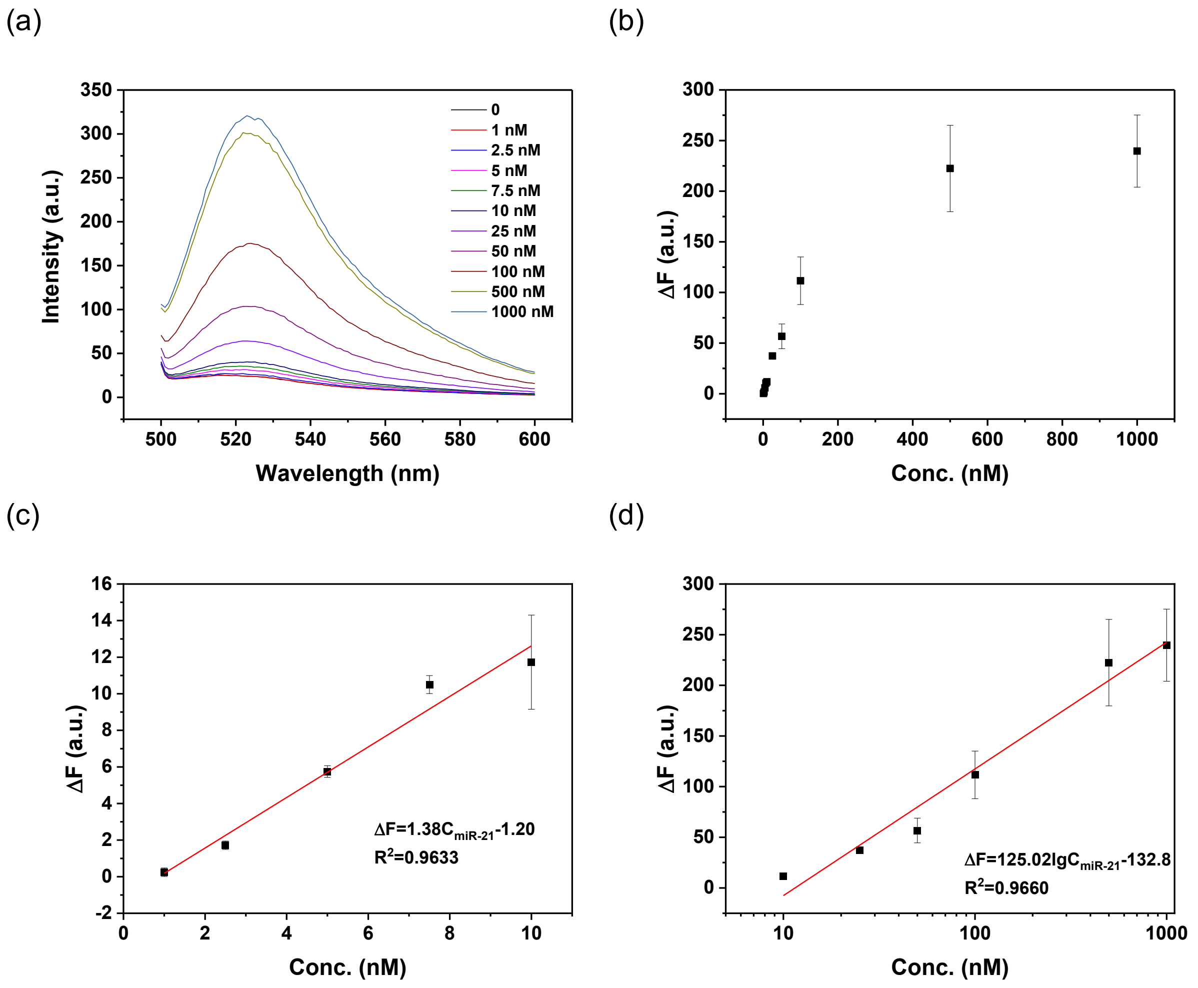

Fig. S7. The detection performance of DFNS-20@DA for miR-21: (a) Fluorescence spectra of DFNS-20@DA and probe complexes for the detection of miR-21 at different concentrations. (b) ΔF versus the concentration of miR-21. (c) Linear correlation between ΔF and C_{miR21}. (d) Linear correlation between ΔF and $logC_{miR21}$

Supplementary Table

Table S1. Comparison of different "turn-on" fluorescent biosensors for DNA and miRNA detection using different solid phase quenchers.

Quencher	Target	LOD	Detection range	Time	Ref.
Organic framework nanoparticles (ZrMOF)	miR-21/DNA	11 aM	10^{-4} -10^{6} pM	30 min	4
Reduced graphene oxide (rGO)	miR-451a	1 nM	1-100 nM	45 min	5
Graphene oxide (GO)	miR-21/DNA	/	10-350 nM	30 min	6
Covalent organic frameworks (COFs)	telomere DNA	50 pM	0.05-10 nM	30 min	7
Polydopamine nanoparticles (PDANs)	miR-21/DNA	0.4 nM	0.1-50 nM	35 min	8
DFNS@DA	miR-21/DNA	0.53 nM	1-1000 nM	20 min	This work

“*” indicates the total time of the fluorescent probe adsorption with quencher and the probe hybridization with target.
“/” stands for ‘‘not reported’’.

References

1. Stöber, W.; Fink, A.; Bohn, E., Controlled Growth of Monodisperse Silica Spheres in the Micron Size Range. *J Colloid Interface Sci* **1968,** *26*, 62-69.
2. Du, X.; Li, X.; Huang, H.; He, J.; Zhang, X., Dendrimer-Like Hybrid Particles with Tunable Hierarchical Pores. *Nanoscale* **2015,** *7*, 6173-6184.
3. Hong, Y.; Yao, Y.; Zhao, H.; Sheng, Q.; Ye, M.; Yu, C.; Lan, M., Dendritic Mesoporous Silica Nanoparticles with Abundant Ti4+ for Phosphopeptide Enrichment from Cancer Cells with 96% Specificity. *Anal Chem* **2018,** *90*, 7617-7625.
4. Liu, S.; Huo, Y.; Fan, L.; Ning, B.; Sun, T.; Gao, Z., Rapid and Ultrasensitive Detection of DNA and Microrna-21 Using a Zirconium Porphyrin Metal-Organic Framework-Based Switch Fluorescence Biosensor. *Anal Chim Acta* **2022,** *1192*, 339340.
5. Xiong, X.; Dang, W.; Luo, R.; Long, Y.; Tong, C.; Yuan, L.; Liu, B., A Graphene-Based Fluorescent Nanoprobe for Simultaneous Imaging of Dual Mirnas in Living Cells. *Talanta* **2021,** *225*, 121947.
6. Pan, W.; Liu, B.; Gao, X.; Yu, Z.; Liu, X.; Li, N.; Tang, B., A Graphene-Based Fluorescent Nanoprobe for Simultaneous Monitoring of Mirna and Mrna in Living Cells. *Nanoscale* **2018,** *10*, 14264-14271.
7. Ahmed, L. R.; Gilmanova, L.; Pan, C.-T.; Kaskel, S.; El-Mahdy, A. F. M., Hollow Spherical Covalent Organic Frameworks from Nonplanar or Planar Monomers for the Fluorescence Detection of Telomere DNA: Role of the 2-(2-Azidoethoxy)Ethoxy Group. *ACS Appl Polym Mater* **2022,** *4*, 9132-9143.
8. Meng, Y.; Liu, P.; Zhou, W.; Ding, J.; Liu, J., Bioorthogonal DNA Adsorption on Polydopamine Nanoparticles Mediated by Metal Coordination for Highly Robust Sensing in Serum and Living Cells. *ACS Nano* **2018,** *12*, 9070-9080.
